# Melatonin in Epilepsy: A New Mathematical Model of Diurnal Secretion

**DOI:** 10.1155/2016/3861461

**Published:** 2016-07-13

**Authors:** Justyna Paprocka, Marek Kijonka, Marcin Pęcka, Maria Sokół

**Affiliations:** ^1^Department of Paediatrics and Developmental Age Neurology, School of Medicine in Katowice, Medical University of Silesia, Ulica Medyków 16, 40-752 Katowice, Poland; ^2^Department of Medical Physics, Maria Skłodowska-Curie Memorial Cancer Center and Institute of Oncology, Gliwice Branch, Ulica Wybrzeże Armii Krajowej 15, 44-101 Gliwice, Poland; ^3^Faculty of Automatic Control, Electronics and Computer Science Biomedical Engineering, Silesian University of Technology, Ulica Akademicka 2A, 44-100 Gliwice, Poland

## Abstract

*Purpose*. The main objective of the study was to create a mathematical model that describes the melatonin circadian secretion and, then the functionality of the model was tested by a comparison of the melatonin secretions in children with and without epilepsy.* Material and Methods*. The patients were divided into the epilepsy group (EG, *n* = 52) and the comparison group (CG, *n* = 30). The melatonin level was assessed by a radioimmunoassay method. The diurnal melatonin secretion was described using a nonlinear least squares method. Spearman's rank correlation coefficient was chosen to estimate the dependence of the acquired data. The model reproduces blood concentration profiles and its parameters were statistically analyzed using the Mann-Whitney-Wilcoxon test and logistic regression.* Results*. The correlation analysis performed for the EG and CG groups showed moderate correlations between age and the melatonin secretion model parameters. Patients with epilepsy are characterized by an increased phase shift of melatonin release.

## 1. Introduction

A complex interaction between sleep and epilepsy is still a matter of debate [1–4]. Sleep deprivation may activate epileptiform activity. Epilepsy* per se* and antiepileptic treatment may cause sleep deprivation or fragmentation causing the vicious circle [1–4]. For some time melatonin has been recommended for children with epilepsy due to its ability to promote sleep and to avoid sleep deprivation [5, 6]. Melatonin, as a hormone, is secreted by pineal gland and its production is regulated by light and retinohypothalamic tract [7–11]. Melatonin secretion depends on the age, as the highest values reported of its concentration are detected between 1 and 7 years of age [2, 9, 12].

Melatonin secretion in epilepsy shows diverse results: higher nocturnal melatonin concentrations, higher melatonin concentration after seizures, or loss of the characteristic diurnal rhythm of secretion.

In animal models the majority of data suggest anticonvulsant properties of melatonin. In human studies it is difficult to reach unambiguous conclusions [1–12].

According to van Golde et al., Coppola et al., and Gupta et al. [5–7] melatonin may improve sleep in people with epilepsy, especially in those with mental retardation. Recent two trials, the first study of melatonin administration conducted on 23 children with refractory epilepsy [5] and the second double-blind, cross-over, placebo controlled trial in 18 children and young adults with epilepsy and mental retardation, did not confirm melatonin influence on seizures' reduction [6]. However, the opposite observation has been reported by Waldhauser et al. [13]. Also our experimental studies have shown circadian seizures pattern to be not proven in humans [1].

Such diverse results indicate that there is still a need for further studies of melatonin secretion in different disorders. The goal of this paper is to develop a mathematical model of the melatonin circadian secretion, to determine its dependency on the clinical features, as well as the differences in secretion patterns in children with and without epilepsy. Multiple mathematical approaches have been proposed to describe circadian melatonin secretion rhythms [14–16]. Most of these methods depend on curve-fitting of the melatonin profile and/or the crossing of a threshold to determine phase. Thus, these methods assume certain physiological characteristics about the shape and amplitude of the melatonin profile. Our approach aims to construct a simple tool that will be helpful in practical clinical applications.

## 2. Materials and Methods

The study was approved by Ethic Committee of Medical University of Silesia. The informed written consent was taken from the parents or caregivers. The study was carried out at the Pediatrics and Developmental Age Neurology Department of Medical University of Silesia in Katowice.

None of the studied subjects had taken any medications affecting melatonin secretion (benzodiazepines and their agonists, fluvoxamine, caffeine, vitamin B12, nonsteroidal anti-inflammatory drugs, aspirin, ibuprofen, indomethacin, alpha-adrenolytics, prostaglandin inhibitors, calcium channel blockers, dexamethasone, and clonidine) before and during the study. Melatonin hypersecretion may be provoked by antidepressants like desipramine, fluvoxamine, and monoamine oxidase inhibitors and that is why patients on such treatment were excluded from the study.

The blood samples were drawn every 3 hours through an intravenous catheter. During night hours, blood samples were taken by red dim light. The melatonin level was determined using radioimmunoassay method (RIA).

### 2.1. Epilepsy Group (EG)

The epilepsy group included 52 patients at the mean age of 6 years and 9 months. Patients with epilepsy were reviewed for the following: seizure type and syndrome, seizure frequency, age at seizure onset, electroencephalogram tracings, current and previous AEDs, seizure timing, etiology, cognitive status, and family history (Table 1).

The type of epileptic seizures was defined following the International League Against Epilepsy Classification and Terminology. Children were divided into two subgroups: with focal seizures (*n* = 26, 50%) and with generalized seizures (*n* = 26, 50%). 13 children from each group have symptomatic epilepsy. Antiepileptic drugs used were as follows: valproic acid (*n* = 38, 72.9%), carbamazepine (*n* = 27, 52.7%), lamotrigine (*n* = 26, 50%), topiramate (*n* = 24, 45.9%), vigabatrin (*n* = 16, 31%), and levetiracetam (*n* = 10, 18.9%).

### 2.2. Comparison Group (CG)

The comparison group constituted of 30 nonepileptic patients at the mean age of 6 years and 11 months. Among these patients tension headache (*n* = 11, 36.7%), peripheral nerve palsies (facial nerve palsy *n* = 11, 36.7%; peroneal nerve palsy, *n* = 3, 10%), and back pain (*n* = 5, 16.7%) were diagnosed. The routine laboratory test and EEG tracing were normal. All children were drug naïve. Because the abovementioned conditions are not reported as influencing endogenous melatonin secretion levels, the inclusion criteria should not affect the results of the melatonin profiles.

## 3. Methods

### 3.1. Mathematical Model

The following function was constructed and applied to approximate output MLT (*t*), the absolute melatonin concentration at time *t* [pg/mL] in patients:

(1) MLTt=b1+b2exp⁡−cos⁡π/12t−π/12b3−12cos⁡π/24b4−1/ln⁡22.

The parameters *b*_1_–*b*_4_ of the secretion function (1) have their biophysical and clinical significance, which are as follows:

 
*b*_1_: minimum melatonin concentration [pg/mL].
 
*b*_2_: melatonin release amplitude [pg/mL].
 
*b*_3_: phase shift of melatonin release [h].
 
*b*_4_: sleep duration (represented by the full width at half maximum (FWHM) of the melatonin secretion model) [h].

Maximum melatonin concentration, *b*_max_ [pg/mL], is given by a sum of *b*_1_ and *b*_2_.

The proposed mathematical model adopts a Gaussian function, as it is often used to describe secretory events [17]. The modeling process starts from the following bell-shaped function:

(2) $$\begin{array}{c} f\left(\;t\;\right)=\exp\left(\;-{\left(\;t-1\;\right)}^{2}\;\right). \end{array}$$

Then, the argument of the model is looped using cosine function and its primitive period is optimized to 24 hours and phase-shifted by a factor *b*_3_:

(3) $$\begin{array}{c} f\left(\;t\;\right)=\exp\left(\;-{\left(\;\cos\left(\;\frac{\pi }{12}t-\frac{\pi }{12}b_{3}\;\right)-1\;\right)}^{2}\;\right). \end{array}$$

The obtained function is periodic (period equals 24 hours) and normalized to 1 in the point:

(4) $$\begin{array}{c} \frac{\pi }{12}b_{3}. \end{array}$$

In the next step the model is vertically scaled and shifted by the factors *b*_2_ and *b*_1_, respectively:

(5) $$\begin{array}{c} f\left(\;t\;\right)=b_{1}+b_{2}\exp\left(\;-{\left(\;cos\left(\;\frac{\pi }{12}t-\frac{\pi }{12}b_{3}\;\right)-1\;\right)}^{2}\;\right). \end{array}$$

Finally, the function is scaled horizontally using expression (6) calculated under the assumption that the factor *b*_4_ is the full width at half maximum of the model (see (1)):

(6) $$\begin{array}{c} {\left(\;\frac{\cos\left(\left(\pi /24\right)b_{4}-1\right)}{\sqrt{\ln2}}\;\right)}^{2}. \end{array}$$

**Figure 1:**
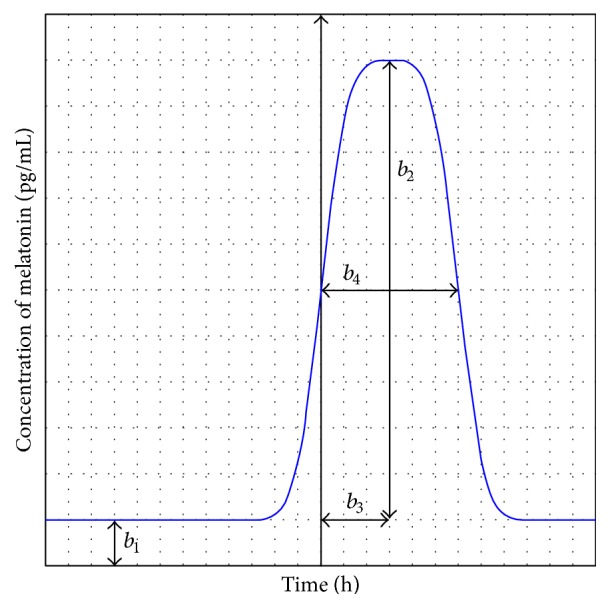
Melatonin secretion model. Solid line: bell-shaped function modeling melatonin secretion; *b*_1_: minimum melatonin concentration; *b*_2_: melatonin release amplitude; *b*_3_: phase shift of melatonin release; *b*_4_: sleep duration (the full width at half maximum, FWHM).

Figure 1 presents the graphical representation of the melatonin secretion model and visualizes the biophysical meaning of its parameters.

## 4. Statistical Analysis

In order to describe the circadian rhythm of melatonin secretion nonlinear least squares estimation was applied. The Levenberg-Marquardt algorithm was chosen to adjust the model parameters that yield the best approximation of the data points for each patient; thus, the shape of the fitted curve was modeled to resemble a natural course of melatonin secretion. The parameters obtained in the fitting were interpreted in terms of their biophysical and clinical meaning and analyzed statistically using the Mann-Whitney-Wilcoxon, *χ* ^2^ Pearson tests and Spearman's rank correlation to find the relationship with clinical features. The *p* values less than 0.05, a predetermined significance level, were accepted as indicating that the observed result would be highly unlikely under the null hypothesis. In case of dichotomous variables of clinical features, the parameters were analyzed using logit regressions. The patients for whom the melatonin secretion curve was not bell shaped (5 cases) were excluded from the analysis.

## 5. Results

The epileptic encephalopathy group (EG) and the comparison group (CG) were homogeneous, as regards age (*p* = 0.7654, Mann-Whitney *U* test), sex (*p* = 0.6871, *χ* ^2^ Pearson), and intellectual development (*p* = 0.6798, Mann-Whitney *U* test).

**Figure 2:**
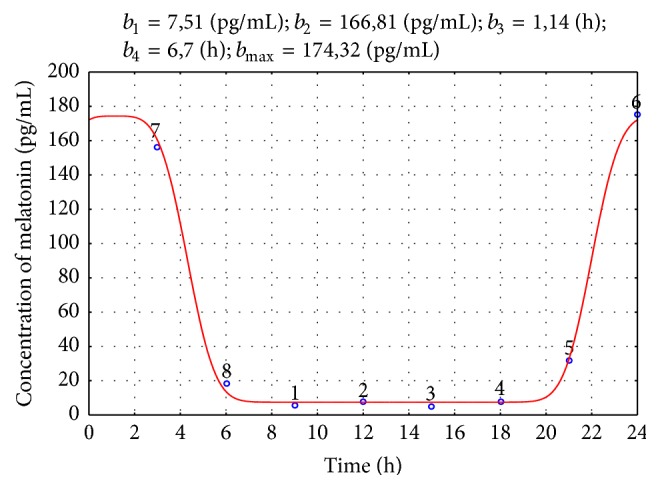
Melatonin secretion for a child from the CG group: solid line: estimated model; circles: measured values. The approximated parameters' values are given above the figure.

**Figure 3:**
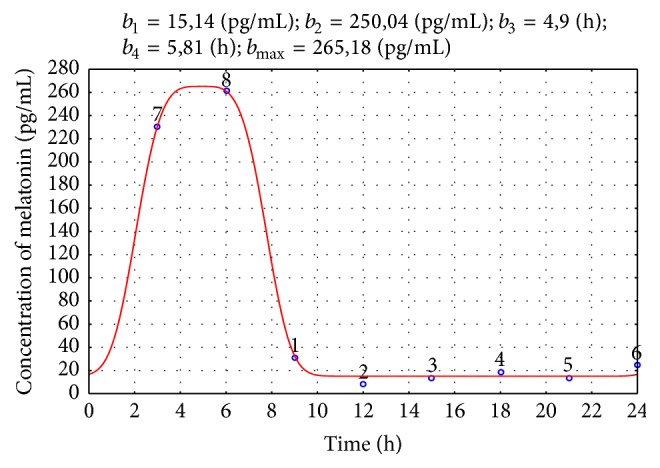
Melatonin secretion for a child from the EG group: solid line: estimated model; circles: measured values. The approximated parameters' values are given above the figure.

The quality of the obtained models was verified by the normality test of the residuals' distribution, statistical significance of the estimated parameters, percentage of the explained variance (>90%), and the *R*-value (>0.95). The illustrative examples of the models obtained for the CG and EG groups are shown in Figures 2 and 3, respectively.

### 5.1. Statistical Comparisons

The parameters' estimates obtained for the melatonin secretion models were subjected to statistical analysis in order to compare the EG group and the CG group. The Mann-Whitney *U* test was applied and the results are presented in Table 2.

**Figure 4:**
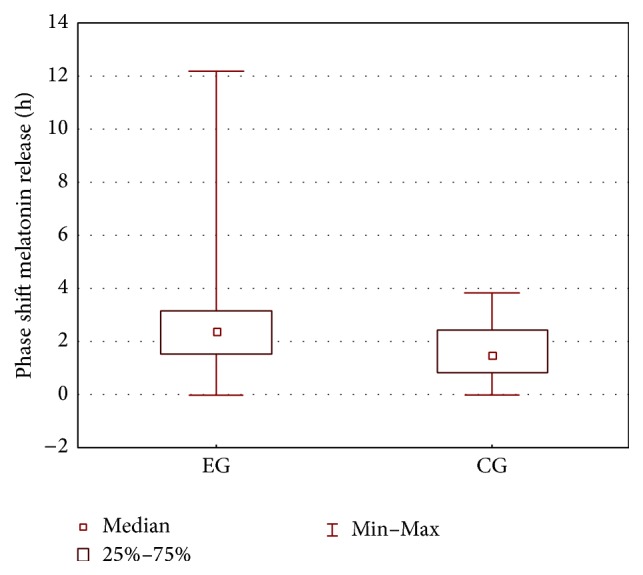
Box plot of the phase shift of melatonin release for the EG and CG groups.

From the comparison of the CG and EG groups (Table 2) it reveals that the only model parameter that differentiates the groups is *b*_3_. The phase shift of melatonin release is increased in the EG group; the box plot of the phase shift of melatonin release for the EG and CG groups is shown in Figure 4.

### 5.2. Correlation Analysis

The Spearman's rank correlation coefficients were calculated to determine correlations between the model parameters:

(i) Minimum melatonin concentration (*b*_1_).
(ii) Melatonin release amplitude (*b*_2_).
(iii) Phase shift of melatonin release (*b*_3_).
(iv) The full width at half maximum (*b*_4_).
(v) Maximum melatonin concentration (*b*_1_ + *b*_2_).

And they were calculated to determine correlations between the clinical features:

- Age.
- Quotient of intellectual development.
- The average number of seizures per day.
- The number of seizures on the day before the day of sampling.
- The number of seizures on the day of sampling (seizures experienced during the 24 hours of sampling).
- Number of antiepileptic drugs.
- Duration of the disease.
- The time (hours) elapsed between the last crisis and the beginning of sampling.

The results obtained for EG and CG are presented in Tables 3 and 4, correspondingly. The variables associated with the disease were analyzed only for the EG group.

### 5.3. Minimum Melatonin Concentration

All the correlation coefficients between *b*_1_ and the clinical features are statistically insignificant (Tables 2 and 3); thus, it may be concluded that *b*_1_ is independent of the investigated clinical features.

### 5.4. Melatonin Release Amplitude

For both CG and EG groups the release amplitude values decrease with age (Tables 2 and 3). Moreover, for the EG group, clinical features of seizures statistically significantly affect the melatonin release amplitude. The amplitude is higher when the seizures occurred just before blood taking and when the number of seizures on the examination day is high (Table 4).

Another factor markedly influencing the release amplitude is duration of the disease: the longer the disease duration, the lower the amplitude (Table 4).

Other dependencies obtained for this parameter (Tables 3 and 4) are not statistically significant.

### 5.5. Phase Shift of Melatonin Release

As seen from Table 4 the phase shift of melatonin release, *b*_3_ parameter, is for EG significantly correlated with the features of the epileptic symptoms (average number of seizures per day, number of seizures on the day before examination and on the day of examination, and time elapsed between the last seizure and the beginning of sampling). With the increase of the epileptic seizures frequency, phase of melatonin release shifts more strongly. It is also worth noting that the number of antiepileptic drugs is found to delay melatonin release time.

For both EG and CG this parameter is statistically independent of the quotient of intellectual development and for EG of the duration of the disease (Tables 3 and 4). In the CG group (Table 3) with age melatonin release is shifted to the later hours.

### 5.6. Sleep Duration: The Full Width at Half Maximum of Melatonin Secretion Model

None of the calculated correlation values obtained for *b*_4_ in both CG and EG groups (Tables 3 and 4) is statistically significant.

### 5.7. Maximum Melatonin Concentration

The Spearman's rank correlation test shows for both CG and EG (Tables 3 and 4) a significant relationship between age and the maximum melatonin concentration (*b*_1_ + *b*_2_). Moreover, in the EG group duration of the disease has an impact on maximum melatonin concentration (negative correlation). Melatonin maximum concentration depends on the number of seizures and the time when they occurred. Maximum concentration of melatonin is found to increase with the number of seizures and the seizure occurrence just before the blood taking.

### 5.8. Analysis of the Responses to the Binary Predictor

The parameters of the created mathematical model (*b*_1_, minimum melatonin concentration, *b*_2_, melatonin release amplitude, *b*_3_, phase shift of melatonin release, and *b*_4_, the full width at half maximum) were used in the logit regression as explanatory variables to predict a logistic response of binary variables (the presence of sleep disorders, the presence of seizures associated with sleep, and predominant type of seizures).

Maximum melatonin concentration was omitted in this statistical classification, as it is defined by a sum of *b*_1_ and *b*_2_.

Logistic regression analysis was carried out only in the EG group, since in the CG group no positive values of dichotomous clinical features are present.

The results of the logistic regression show no statistically significant dependencies between binary variables and the model parameters are observed.

## 6. Discussion

The vast majority of melatonin studies were focused on the effectiveness of administration of this hormone in sleep disturbances, also in case of epileptic patients [17–26].

Our study aimed to compare the melatonin rhythms in subjects with and without epilepsy. To the best of our knowledge, this is the first study in which the circadian melatonin levels of epileptic patients (EG) and children without epilepsy (CG) have been compared and modeled mathematically. The model of melatonin secretion provides a set of parameters directly characterizing melatonin cycle, such as minimum melatonin concentration, melatonin release amplitude, phase shift of melatonin release, and estimated sleep duration, which could be of potential clinical usefulness as the factors facilitating classification of sleep disturbances.

The constructed function was assumed to reflect physiological rhythm of melatonin concentration changes (periodicity and monotonicity) and its shape-modeling parameters are in good agreement with the physiological terms as confirmed by applying rigorous restrictions on the quality of approximation (*R*-value > 0.95). In most cases (for >80% of the investigated population) the percentage of the explained variance exceeds 98%.

The main limitation of this approach is its restriction to the bell-shaped circadian rhythms (the most characteristic release). However, even in case of non-bell-shaped melatonin secretion or when endogenous periods of circadian rhythms deviate from 24 h, the approximated model parameters may be useful as an indicator of a lack of melatonin release (similar values of minimum concentration and maximum concentration). In case of physiologically disturbed melatonin secretion (double release, non-bell-shaped circadian rhythms) the received parameters of the phase shift of melatonin release and FWHM may be incorrect. If melatonin levels fluctuate over the course of a day or during ejection, the parameters of the obtained melatonin secretion models may also be affected; in such case decrease of the fit goodness may indicate deviations from the physiological course. This provides another important information on secretion disturbances of melatonin circadian rhythm.

Term (6) (sleep duration) is obtained under the assumption that the full width at half maximum equals *b*_4_ and for *b*_4_ > 0 and *b*_4_ < 12 the minimum of function (1) equals approximately *b*_1_, since the exponential part is close to zero.

Because the secretion models should reflect physiologically realistic situations, the modeling algorithm is constructed in such a way that it allows only the values from the following ranges: *b*_1_ ∈ [0, +*∞*); *b*_2_ ∈ [0, +*∞*); *b*_3_ ∈ [0,24); *b*_4_ ∈ (0,12).

Our model aims mainly at modeling the circadian secretion rhythms and in case of longer time intervals (48 hours or more) extra parameters should be used, for example, to characterize the distance between successive melatonin releases or the amplitude changes of different melatonin maxima.

The results of logistic regression were found to be statistically insignificant in our study. Though, the ratio of the group size to the number of predictors equals 13, thus, the requirement of the logit regression analysis is fulfilled (this ratio should be larger than 10), but it may be expected that increasing size of the EG group will provide a better insight into melatonin patterns. It will also help to obtain normal distributions of the variables and allow parametric analysis to be applied to detect more subtle changes in the datasets.

In healthy subjects' serum the melatonin concentration peak occurs between 2 am and 4 am; then it gradually declines [2]; however melatonin release may be shifted with time zone due to its day and night light dependence. In the current study, age and peak serum melatonin level are negatively correlated for the EG and CG groups, whereas a phase shift of melatonin release depends positively on age in the CG group.

In case of epileptic subjects, the situation is much more complicated. Circadian clock is expected to play an important role in epileptic patients; however, still relatively little is known. The effects of epilepsy on secretion processes of melatonin and vice versa have been described in several papers presenting, however, wildly conflicting results. Some authors report low baseline levels of melatonin in people with epilepsy [27, 28], whilst others observe elevated levels with a maintained day-night rhythm [29] or with a phase difference compared to controls [13]. On the other hand, normal plasma melatonin curve in epilepsy patients under dim lit conditions [30] as well as in the study involving epileptic children [3] was found.

Our study performed on the homogeneous, as regards age, sex, and intellectual development, groups points to the role of seizures in melatonin cycle disturbances. In epileptic patients, the phase shift of melatonin release occurs later as compared to the CG group, and the correlation study indicates that there is a relationship between a phase shift of melatonin peak and the seizures (positive correlation). Interestingly, the age dependencies in the EG group reveal similar trends as for CG (i.e., negative correlations between age and the amplitude of melatonin release as well as age and maximum melatonin concentration), which seems to confirm validity of the model.

Melatonin concentration in patients with epilepsy is usually claimed to be slightly increased or unchanged as compared to normal values [27–30], which is also generally confirmed by our observations. However, there is a statistical dependence between melatonin release amplitude and the number of seizures in different time intervals in the EG group. Moreover, the time since last seizure has a significant effect on the secretion of melatonin. Duration of the disease is also found to influence the obtained circadian melatonin cycle in the EG group. It should be noted that antiepileptic treatment itself may affect melatonin secretion, which, in fact, is seen in our correlation analysis. For example, valproic acid, due to its interaction with GABA transmission at suprachiasmatic nucleus, may lower melatonin secretion [22]. On the other hand some antiepileptic drugs like lamotrigine and levetiracetam may have positive effect on the sleep structure resulting in more REM and slow-wave sleep [31]. The direct effect of AEDs on sleep is difficult to measure because of many confounding factors, with the leading one, polypharmacy [31, 32].

Also oral melatonin supplementation effects need further studies [33, 34]. During the last decade, melatonin has started to be considered as an attractive option in order to minimize the neurological sequelae from hypoxic-ischemic brain injury. The brain itself is particularly sensitive to free radicals damage due to its high utilization of oxygen, its relatively poorly developed antioxidant defense, and its high amount of easily oxidizable fatty acids. Antioxidant properties of melatonin may have positive effect on children with epilepsy.

## 7. Conclusions

Hormonal secretion is a very complex physiological process and modeling studies, though they usually focus on a particular step or pathway involved in this phenomenon, may be helpful in getting clinical insight into the overall dynamics of the operating processes.

Mathematical modeling of circadian melatonin cycle facilitates statistical analysis of the patients' hormone levels offering a set of parameters that enable objectification of the secretion description.

## Figures and Tables

**Table 1:** Clinical characteristics of epilepsy group (EG).

	Epilepsy with focal seizures *N* = 26	Epilepsy with generalized seizures: Lennox-Gastaut syndrome *N* = 26
Epilepsy duration (months)	11–200 months	12–194 months

Epilepsy etiology	Unknown etiology *N* = 13, 50% Known etiology *N* = 13, 50% (cortical dysplasia)	Unknown etiology *N* = 13, 50% Known etiology *N* = 13, 50%

The number of antiepileptic drugs (mean)	1–4 (2)	1–3 (2)

The frequency of seizures per day (mean)	1–9 (2)	1–6 (1)

The number of seizures on the day before the day of sampling (mean)	1–3 (1)	1–3 (1)

The average number of seizures on the day of sampling	1	1

The number of hours elapsed between the last seizure and the beginning of the sampling (mean)	1–42 (12.5)	1–42 (12.4)

**Table 2:** The results of Mann-Whitney *U* test for the CG and the EG groups. The results marked in bold are significant with *p* < 0.05.

	Median of the EG group	Median of the CG group	*p* value
Minimum melatonin concentration [pg/mL]	5.9933	6.4645	0.8813
Melatonin release amplitude [pg/mL]	117.9186	142.7516	0.0822
Phase shift of melatonin release [h]	**2.3851**	**1.4843**	**0.0071**
Estimated sleep duration [h]	7.2894	7.1835	0.522
Maximum melatonin concentration [pg/mL]	124.7568	151.8289	0.1068

**Table 3:** Spearman's rank correlation coefficients calculated for the CG group. The statistically significant correlations (*p* < 0.05) are marked in bold.

	Minimum melatonin concentration, *b*_1_	Melatonin release amplitude, *b*_2_	Phase shift of melatonin release, *b*_3_	Estimated duration of sleep, *b*_4_	Maximum melatonin concentration, *b*_max_
Age	0.0258	−**0.4463**	**0.422**	0.1117	−**0.4443**
Quotient of intellectual development	−0.047	−0.0865	0.1222	−0.2476	−0.087

**Table 4:** Spearman's rank correlation coefficients calculated for the EG group. The statistically significant correlations (*p* < 0.05) are marked in bold.

	Minimum melatonin concentration, *b*_1_	Melatonin release amplitude, *b*_2_	Phase shift of melatonin release, *b*_3_	Estimated duration of sleep, *b*_4_	Maximum melatonin concentration, *b*_max_
Age	0.1717	−**0.2894**	0.0966	−0.1704	**−0.2694**
Quotient of intellectual development	−0.1046	0.1315	0.2065	0.1579	0.107
Average number of seizures per day	−0.1871	**0.3863**	**0.3171**	0.2454	**0.36**
The number of seizures on the day before the day of sampling	−0.1287	**0.2736**	**0.3655**	0.1898	0.2719
The number of seizures on the day of sampling	−0.1463	0.244	**0.4306**	0.1619	0.238
Number of antiepileptic drugs	−0.0731	0.0142	**0.2922**	−0.1203	0.0121
Epilepsy duration	−0.00427	−**0.3608**	0.1393	−0.1569	−**0.3468**
Hours elapsed between the last seizure and the beginning of sampling	0.02774	−**0.33**	−**0.5012**	−0.1668	−**0.3293**
